# Late-Onset Pulmonary Artery Stump Thrombosis 13 Years After Lobectomy: A Case Report

**DOI:** 10.7759/cureus.97416

**Published:** 2025-11-21

**Authors:** Jake Belli, Philopateer Messeha, Edmond Mala, Gaurav Ahluwalia

**Affiliations:** 1 Medicine, Lake Erie College of Osteopathic Medicine, Bradenton, USA; 2 Hospital Medicine, AdventHealth Florida, New Smyrna, USA

**Keywords:** anticoagulation, deep vein thrombosis, lobectomy, pulmonary artery stump thrombosis, squamous cell carcinoma

## Abstract

Pulmonary artery stump thrombosis (PAST) is a rare complication that forms after partial or complete resection of the lung. It involves the formation of a blood clot at the ligated end of the pulmonary artery, which forms due to stasis in the residual vessel. We report a case of PAST in an 83-year-old male lung cancer survivor, status post right middle and lower lobectomy in 2012, who presented with symptoms of generalized weakness and repeated falls. PCR tests were positive for rhinovirus and parainfluenza virus, and he was started on prednisone. A CT scan of the chest showed thrombosis at the ligated right pulmonary artery. The patient was administered a therapeutic dose of enoxaparin (Lovenox) and was discharged two days later. Despite presenting with certain risk factors, the patient was not discharged with anticoagulation medication.

## Introduction

Surgical resection of lung cancer is a well-known risk factor for venous thromboembolism (VTE) due to endothelial injury and immobility. However, a less common manifestation of VTE is known as pulmonary artery stump thrombosis (PAST), which can occur as a complication following lobectomy or pneumonectomy. PAST occurs when a thrombus is formed at the ligated end of the pulmonary artery, which can potentially lead to serious complications such as pulmonary infarction or embolization. The estimated incidence of PAST following pneumonectomy is 12%, and thrombus formation is more likely to occur in the right stump [1]. While PAST is usually an incidental finding on computed tomography (CT), it must be clinically differentiated from a pulmonary embolism because of their different prognoses and treatments. PAST is typically confined to the post-surgical vascular stump and appears as a clinically silent concave defect on CT. In contrast, a pulmonary embolism extends distally into the pulmonary arteries, often presenting with cardiopulmonary symptoms, and is more often convex on CT [1]. Most clinicians regard PAST as a benign entity due to its incidental findings and therefore can opt against the anticoagulation route of treatment. However, a case report linked PAST occurring 10 years after right pneumonectomy with multiple pulmonary emboli and pulmonary hypertension [2]. These clinicians opted for prolonged postoperative anticoagulation and suggested anticoagulation in all patients undergoing pneumonectomy. The potential for severe late complications, especially when occurring years after surgery, highlights the importance of selective anticoagulation in high-risk cases. Here, we present a case of an 83-year-old male with a late-onset, incidental PAST finding attributed to right middle and lower lobectomy secondary to squamous cell carcinoma who did not undergo long-term anticoagulation.

## Case presentation

An 83-year-old male presented to the emergency department with complaints of generalized weakness and repeated falls over the past five days. His past medical history includes stage IIB squamous cell carcinoma of the lung, which was diagnosed in 2011 and treated with right middle and lower lobectomy followed by chemoradiation in 2012. He also has a history of chronic obstructive pulmonary disease (COPD), type 2 diabetes mellitus, and idiopathic atrial fibrillation treated with diltiazem. On admission, white blood cells (WBC) were 15,650 /mm^3^, hemoglobin was 11.4 g/dL, troponin was 30 ng/dL, lactic acid was 2.50 mmol/L, oxygen saturation was 97%, and blood pressure was 98/75 mmHg. His laboratory findings are shown in Table 1. He was admitted to the hospital for sepsis, and upon workup, diagnostic tests were positive for rhinovirus and parainfluenza virus, which prompted the administration of prednisone.

**Table 1:** Initial laboratory findings HbA1c: glycated hemoglobin; eGFR: eGFR: estimated glomerular filtration rate; AST: aspartate aminotransferase; ALT: alanine aminotransferase

	Patient’s Lab Values	Age-Adjusted Reference Range
White Blood Cells (/mm^3^)	15,650	3,600 - 11,000
Hemoglobin (g/dL)	11.4	14 - 18
Hematocrit (%)	31.2	41 - 53
Mean Corpuscular Volume (μm^3^)	88	80 - 100
Sodium (mEq/L)	136	136 - 146
Potassium (mEq/L)	4.2	3.5 - 5.0
Chloride (mEq/L)	97	95 - 105
Calcium (mEq/L)	9.5	8.4 - 10.2
Glucose Fasting (mg/dL)	122	70 - 110
HbA1c (%)	6.5	< 6.5
Urea Nitrogen (mg/dL)	33	7 - 18
Creatinine (mg/dL)	1.16	0.6 - 1.2
eGFR (mL/min/1.73m^2^)	62.5	60 - 89
AST (U/L)	12	12 - 38
ALT (U/L)	12	10 - 40
Lactic Acid (mmol/L)	2.50	0.70 - 2.10
N-Terminal Pro-BNP (pg/mL)	1113	< 450
Troponin (ng/dL)	30	< 22
D-dimer (μg/mL)	2.17	0 - 0.45

**Figure 1:**
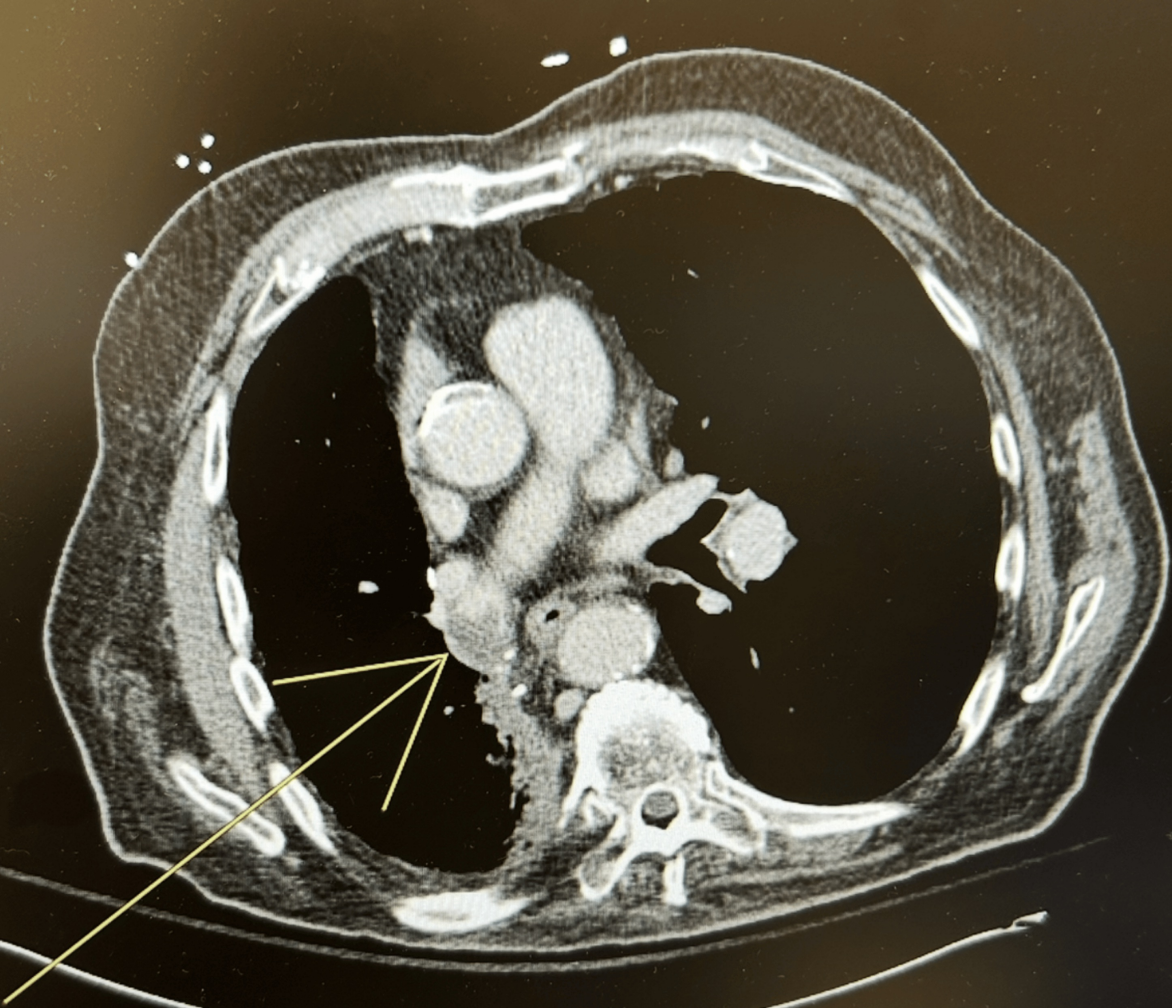
Thin-slice (1 mm) contrast-enhanced axial CT scan showing a concave intraluminal filling defect within the right pulmonary artery stump (yellow arrow). The thrombus measures approximately 15 mm in length by 8 mm in width.

A thin-slice contrast-enhanced CT scan of the chest showed a concave intraluminal thrombus in the right lower lobe measuring approximately 15 mm in length by 8 mm in width, with no evidence of a pulmonary embolus (Figure 1). Despite the patient denying respiratory symptoms, the recommendation of D-dimer and lower extremity Doppler ultrasound was ordered. The D-dimer levels were elevated at 2.17 μg/mL, prompting initiation of therapeutic enoxaparin. Ultrasound studies revealed no deep vein thrombosis in either the right or left lower extremity. A prior chest CT with contrast was performed three months earlier and showed no increase in stump density, suggesting the thrombosis was a new finding. The patient's EKG showed a normal sinus rhythm. The patient’s comprehensive workup did not show any evidence for underlying malignancy, relapse of prior malignancies, or any abnormal coagulation panel results. The patient was discharged two days after the incidental finding without a prescription for home anticoagulation. He was advised to follow up with pulmonology in six to eight weeks for repeat chest CT and pulmonary function testing.

## Discussion

PAST is considered a rare phenomenon due to its unclear clinical implications. It is frequently identified incidentally on postoperative imaging, but the lack of existing literature poses an important question regarding proper management and potential risks. Some cases may remain clinically silent and resolve spontaneously, while other cases may pose a risk for embolic complications or indicate an underlying hypercoagulable state or tumor recurrence [2]. The lack of clarity in the literature warrants further investigation into recent cases of PAST to identify common trends in presentation, anticoagulation, and factors associated with lung resection.

According to a 2022 study that analyzed the epidemiology of PAST, the most common complaints that warranted findings of a thrombosis stump were fever and dyspnea [3]. This study also demonstrated that PAST occurred more frequently following pneumonectomy compared to lobectomy, with a mean time to diagnosis of approximately 4.5 months after surgery. In a study that investigated clinical characteristics following stump thrombosis findings, the median time interval from lung resection to detection of PAST was 3.8 months [4]. These findings highlight the unusual nature of our patient’s presentation, in which PAST was identified on CT imaging 13 years after a right middle and lower lobectomy. Recent case reports have described instances of late-onset PAST occurring several years after lung resection, suggesting that the phenomenon does not have to occur within months of resection. Joshi et al. described a 68-year-old man who developed a right PAST 10 years after pneumonectomy and was treated with heparin followed by warfarin, leading to symptomatic and radiologic improvements [5]. Similarly, Akcam et al. reported a 73-year-old man who developed left PAST three years after pneumonectomy and achieved symptomatic and radiologic improvement following intravenous heparin for three days and oral warfarin [6]. Mirijello et al. described a 59-year-old patient who developed a right PAST four years after pneumonectomy and was started on low molecular weight heparin followed by oral anticoagulation therapy [7]. This combination achieved a near-complete resolution four months after the thrombosis. In contrast, Thomas et al. presented a patient with PAST, which occurred 10 years after lung resection and was complicated by pulmonary hypertension [2]. Despite treatment with anticoagulation, the patient only gained partial resolution due to chronic vascular remodeling. Altogether, these reports show that late-onset PAST can manifest many years after lung resection, often in patients with prior malignancy or other comorbidities that cause vascular remodeling and still have the potential to respond to anticoagulation.

Given the possibility of stump thrombosis recurrence due to underlying hemodynamic factors or malignancy, it is important to radiologically differentiate between PAST and tumor recurrence. On contrast CT, PAST appears as a smooth, concave, low-attenuation filling defect at the vascular stump. In contrast, a recurrent tumor presents as a soft tissue mass that has irregular borders and increased metabolic uptake [8]. The radiological interpretation of PAST versus tumor recurrence is essential because the lack of intervention can cause delayed anticoagulation or oncologic management. The potential for delayed vascular complications emphasizes the possible importance of postoperative surveillance. However, in a 2019 study that focused on patients with resected lung tumors, they followed patients for five years and found that more frequent surveillance imaging was not associated with improved survival or post-recurrence survival [9]. Our case highlights the possibility of vascular complications occurring well beyond the typical surveillance window. This highlights the potential value of prolonged surveillance in certain patients, particularly those with prothrombotic risk factors or evidence of vascular invasion by the tumor [10]. To minimize the incidence of PAST regardless of the timing of its onset, efforts should be made to minimize vascular injury and remove any residual vascular structures [11]. While PAST is mostly considered a benign finding, there have been reports of patients dying of complications related to stump thrombosis despite treatment with anticoagulation [5]. 

Due to the rarity of this condition, there is no definitive protocol on whether to give anticoagulation or not following PAST. A retrospective cohort study between 2011 and 2019 found no difference in the prognosis of patients who received anticoagulation versus those who did not [4]. However, recent case reports describing patients who developed PAST at least one year after lung resection indicate that while all were treated with anticoagulation, follow-up CT scans did not reveal complete resolution of all stump thromboses [7]. The decision to initiate anticoagulation relies on risk factors, which include chest pain, dyspnea, convex-shaped thrombus on CT scan, prior history of venous thromboembolism, late-onset PAST greater than one year after resection, or current underlying malignancy [7]. According to previous reports, the significant time lapse of 13 years from lung resection to PAST development would be a notable risk factor for initiating anticoagulation. However, our patient was discharged after his sepsis resolved and was not administered anticoagulation for home. The clinical reasoning for not initiating at-home anticoagulation was that the lung vasculature was not directly affected. Despite our patient not presenting with the typical cardiopulmonary symptoms of chest pain and dyspnea, he had symptoms relating to generalized weakness and repeated falls. While it is more likely that our patient’s age, comorbidities, and sepsis contributed to the generalized weakness, it cannot be discounted that this is an abnormal presentation of PAST.

## Conclusions

Pulmonary artery stump thrombosis (PAST) is a rare but potentially serious complication following pulmonary resection. Its clinical significance stems from the risk of severe thromboembolic events, which emphasizes the importance of early detection and management. Although dyspnea is the most common presenting symptom, the unusual presentation of generalized weakness in our patient underscores the multifactorial complexity of PAST. The extended interval between lobectomy and presentation in our patient further highlights the atypical nature of this case. While current guidelines for treatment are unestablished, it is important to consider risk factors when deciding to pursue anticoagulation. Further research is essential to develop standardized guidelines on the optimal duration of anticoagulation and whether anticoagulation is necessary to achieve better patient outcomes.

## References

[1] Kim SY, Seo JB, Chae EJ (2005). Filling defect in a pulmonary arterial stump on CT after pneumonectomy: radiologic and clinical significance. AJR Am J Roentgenol.

[2] Thomas PA, Doddoli C, Barlési F, Reynaud-Gaubert M, Giudicelli R, Fuentes P (2006). Late pulmonary artery stump thrombosis with post embolic pulmonary hypertension after pneumonectomy. Thorax.

[3] Gurel Durmus Z, Bulbul Y, Tekinbas C, Seyis KN, Kosucu P (2022). Frequency and predictors of pulmonary arterial stump thrombosis following pneumonectomy or lobectomy. Med Princ Pract.

[4] Park JE, Cha SI, Lee DH (2024). Clinical characteristics and course of pulmonary artery stump thrombosis following lung cancer surgery: a retrospective study from tertiary care hospital. Medicine (Baltimore).

[5] Joshi M, Farooq U, Mehrok S, Srouji N (2009). Delayed formation of pulmonary artery stump thrombus: a case report and review of the literature. Thromb J.

[6] Akcam TI, Kaya SO, Samancilar O, Ceylan KC (2016). Pulmonary artery stump thrombosis developed during the late postoperative period. Kardiochir Torakochirurgia Pol.

[7] Mirijello A, Santoliquido M, Piscitelli P (2021). Pulmonary artery stump thrombosis: to treat or not to treat? The question is still open. Description of a case and review of the literature. Front Cardiovasc Med.

[8] Goto T, Maeshima A, Kato R (2012). Lung adenocarcinoma with peculiar growth to the pulmonary artery and thrombus formation: report of a case. World J Surg Oncol.

[9] McMurry TL, Stukenborg GJ, Kessler LG (2018). More frequent surveillance following lung cancer resection is not associated with improved survival. A nationally representative cohort study. Ann Surg.

[10] Maeda R, Yoshida J, Ishii G (2010). Long-term outcome and late recurrence in patients with completely resected stage IA non-small cell lung cancer. J Thorac Oncol.

[11] Yoon HJ, Kim KH, Jeong MH, Cho JG, Park JC (2019). Very late unusual thrombosis of the remnant pulmonary vasculatures after lung resection complicated by embolic events. J Cardiothorac Surg.

